# Advances in Molecular Diagnosis of Tuberculosis

**DOI:** 10.1128/JCM.01582-19

**Published:** 2020-09-22

**Authors:** Emily MacLean, Mikashmi Kohli, Stefan F. Weber, Anita Suresh, Samuel G. Schumacher, Claudia M. Denkinger, Madhukar Pai

**Affiliations:** a Department of Epidemiology, Biostatistics, and Occupational Health, McGill University, Montreal, Canada; b McGill International TB Centre, McGill University, Montreal, Canada; c Department of Infectious Diseases, University of Heidelberg, Heidelberg, Germany; d Foundation for Innovative New Diagnostics, Geneva, Switzerland; e Manipal McGill Program for Infectious Diseases, Manipal Academy of Higher Education, Manipal, India; Emory University

**Keywords:** accuracy, diagnostics, molecular, tuberculosis

## Abstract

Molecular tests for tuberculosis (TB) have the potential to help reach the three million people with TB who are undiagnosed or not reported each year and to improve the quality of care TB patients receive by providing accurate, quick results, including rapid drug-susceptibility testing. The World Health Organization (WHO) has recommended the use of molecular nucleic acid amplification tests (NAATs) tests for TB detection instead of smear microscopy, as they are able to detect TB more accurately, particularly in patients with paucibacillary disease and in people living with HIV. Importantly, some of these WHO-endorsed tests can detect mycobacterial gene mutations associated with anti-TB drug resistance, allowing clinicians to tailor effective TB treatment. Currently, a wide array of molecular tests for TB detection is being developed and evaluated, and while some tests are intended for reference laboratory use, others are being aimed at the point-of-care and peripheral health care settings. Notably, there is an emergence of molecular tests designed, manufactured, and rolled out in countries with high TB burden, of which some are explicitly aimed for near-patient placement. These developments should increase access to molecular TB testing for larger patient populations. With respect to drug susceptibility testing, NAATs and next-generation sequencing can provide results substantially faster than traditional phenotypic culture. Here, we review recent advances and developments in molecular tests for detecting TB as well as anti-TB drug resistance.

## Abstract (precis)

Molecular tests for tuberculosis (TB) have the potential to help reach the three million people with TB who are undiagnosed or not reported each year and to improve the quality of care TB patients receive by providing accurate, quick results, including rapid drug-susceptibility testing. The World Health Organization (WHO) has recommended the use of molecular nucleic acid amplification tests (NAATs) tests for TB detection instead of smear microscopy, as they are able to detect TB more accurately, particularly in patients with paucibacillary disease and in people living with HIV.

## INTRODUCTION

With an estimated 1.5 million attributable deaths and 10 million new cases in 2018, tuberculosis (TB) is the leading infectious disease killer globally (1). Despite the severity of the epidemic, approximately 3 million people with TB were deemed “missing” due to underdiagnosis as well as underreporting to national TB programs (1). The World Health Organization (WHO) End TB Strategy calls for finding these missing millions in order to meet the sustainable development goal of ending TB by 2030. New diagnostic tests and optimized test deployment strategies will be critical for achieving this target (2). In the context of the ongoing COVID-19 pandemic, it is also important to consider integrating testing for TB and severe acute respiratory syndrome coronavirus 2 (SARS-CoV-2) since symptoms and testing technologies overlap (3).

Over the last decade, the field of TB diagnostics has seen advances in the form of new molecular tests. Often referred to as nucleic acid amplification tests (NAATs), these assays rely on amplification of a targeted genetic region of the Mycobacterium tuberculosis complex, typically by PCR. NAATs can detect TB and perform drug susceptibility testing (DST) for key drugs, such as rifampin (RIF) and isoniazid (INH), more quickly than conventional mycobacterial culture and are also available at different levels of health care systems. As such, they are disrupting the field of TB diagnostics and are helping to improve the quality of TB care (4, 5). Here, we review recent advances in the field of molecular diagnostics for TB and relevant WHO policies and describe the emerging landscape. For advances in biomarker-based tests for active and latent TB detection, we refer the readers to other review articles (6, 7).

## 

### State of the art.

**FIG 1:**
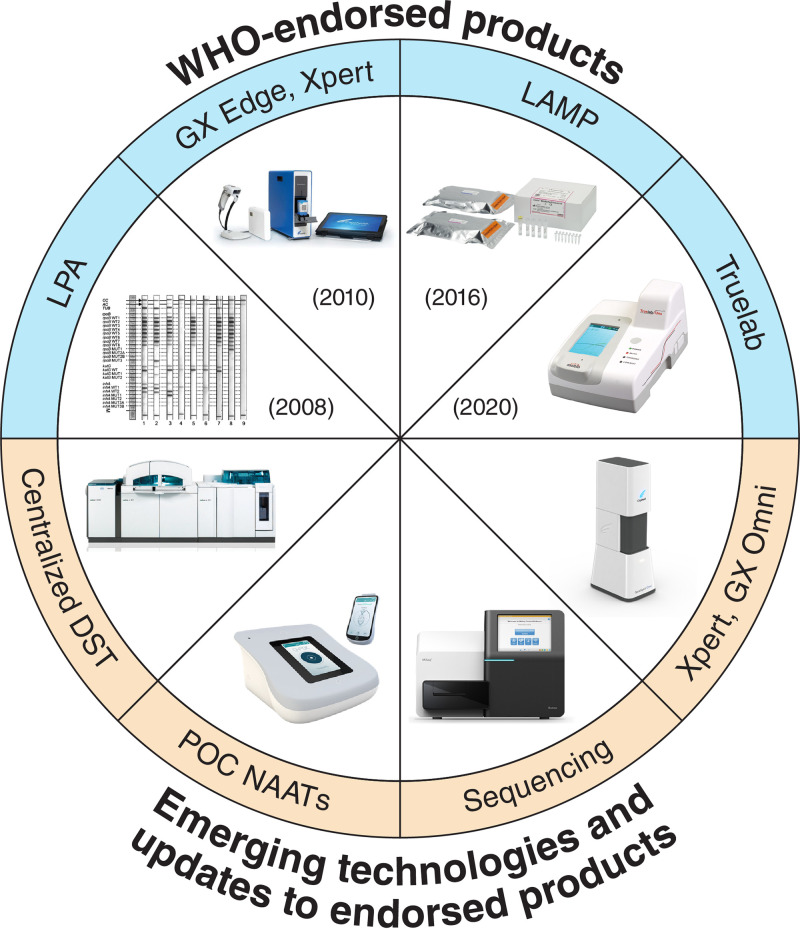
WHO-endorsed and emerging molecular tests for TB and drug resistance. Outlined in blue are WHO-endorsed NAATs, including LPAs (14), Xpert Ultra (20), LAMP (16), and Truelab (21). Tests that are not yet WHO endorsed but are under development or evaluation are outlined in orange. Images shown are examples of products within each category. DST, drug sensitivity testing; GX, GeneXpert; LAMP, loop-mediated isothermal amplification; LPA, line probe assay; POC, point of care; NAAT, nucleic acid amplification test.

As shown in Fig. 1 and Table 1, there are several molecular TB tests that are already WHO recommended and commercially available. Since the Xpert MTB/RIF assay (Cepheid, Sunnyvale, USA) was first endorsed in 2010, advances in the field of TB diagnostics have mostly been in the realm of NAATs and responsive to the needs articulated by published target product profiles (TPPs) (8, 9). More than ever before, new assays are emerging and undergoing validation for TB and TB drug-resistance detection. However, simply developing new tests is insufficient for ensuring their implementation in countries with the highest TB burdens, and barriers to scale-up molecular tests like Xpert MTB/RIF have been identified (10). A 2018 study showed that despite a high diagnostic accuracy and quick time to results, the ratio of smear microscopy tests to Xpert tests performed in 17 countries with a high TB burden was 6 to 1 (11). A similar trend of low uptake of new TB tests has also been reported for urine lipoarabinomannan (LAM) testing (12). For new tests to have impact, they must be adopted and scaled up (13).

**TABLE 1:** WHO-endorsed molecular tests for pulmonary TB detection and drug susceptibility testing^*a*^

Technology	Year endorsed	Method principle	Intended use	Sensitivity (%)^*b*^	Specificity (%)^*b*^	Target setting of use	Turnaround time (h)	Amenable to rapid test-and-treat?	Reference for policy guidance
Xpert MTB/RIF	2010	qPCR	MTB diagnosis and RIF resistance detection	85 (pooled), 96 (RIF resistance)	99 (MTB detection) 98 (RIF resistance)	District or subdistrict laboratory	<2	Yes, especially on Omni platform	WHO 2020 (21), WHO 2016 (84)
Xpert MTB/RIF ultra	2017	qPCR/melting temperature analysis (RIF resistance)	MTB diagnosis and RIF resistance detection	90 (pooled), 94 (RIF resistance)	96 (MTB detection), 98 (RIF resistance)	District or subdistrict laboratory	<2	Yes, especially on Omni platform	WHO 2020 (21)
First-line probe assays (e.g., GenoType MTBDRplus and NIPRO)	2008	PCR, hybridization	Diagnosis of RIF and INH resistance	98 (RIF resistance), 84 (INH resistance)	99 (RIF resistance), >99 (INH resistance)	Reference laboratory	5	No	WHO 2008 (14)
Second-line probe assays (e.g., GenoType MTBDRsl)	2016	PCR, hybridization	Diagnosis of FLQ and SLID resistance	86 (FLQ resistance), 87 (SLID resistance)	99 (FLQ resistance), 99 (SLID resistance)	Reference laboratory	5	No	WHO 2016 (15)
Loopamp MTBC assay	2016	Loop-mediated isothermal amplification	MTB diagnosis	78 (pooled)	98 (MTB detection)	Peripheral laboratory	<2	Yes	WHO 2016 (16)
Truenat MTB plus	2020	Micro RT-PCR	MTB diagnosis	80 (pooled)	96 (MTB detection)	Peripheral laboratory	<2	Yes, on Truelab platform	WHO 2020 (21)
Truenat MTB-RIF Dx	2020	Micro RT-PCR	Diagnosis of RIF resistance	84 (RIF resistance)	97 (RIF resistance)	Peripheral laboratory	<2	Yes, on Truelab platform	WHO 2020 (21)

a FLQ, fluoroquinolone; INH, isoniazid; LAMP, loop-mediated isothermal amplification; NAAT, nucleic acid amplification tests; RIF, rifampin; RT-PCR, reverse transcriptase PCR; SLID, second-line injectable drugs; SSM+/C−, sputum smear microscopy positive/culture positive; SSM−/C+, sputum smear microscopy negative/culture positive; WHO, World Health Organization.

b Performance estimates have been retrieved from different studies and are not the result of head-to-head comparisons. Therefore, comparing performances between tests must be made with caution. All reported values are from the policy guidance document cited.

## DEVELOPMENTS IN TEST AND PLATFORMS WITH WHO ENDORSEMENT

Table 1 provides an overview of all currently available NAATs that are endorsed by WHO, along with information on diagnostic accuracy.

### Line probe assays.

Line probe assays (LPA) for first-line TB drugs (INH and RIF) have been endorsed by WHO for over a decade for the detection of multiple-drug-resistant TB (MDR-TB) (14). These assays include GenoType MTBDRplus (Hain Lifesciences-Bruker, Nehren, Germany) and Nipro NTM+MDRTB II (Osaka, Japan). New-generation LPAs have emerged with higher sensitivity, and some (e.g., GenoType MTBDRsl version 2.0; Hain Lifesciences-Bruker) can detect mutations associated with fluoroquinolones (FLQs) and second-line injectables, kanamycin, amikacin, and capreomycin, and are recommended to guide MDR-TB treatment initiation (15).

### Loop-mediated isothermal amplification.

Loop-mediated isothermal amplification (LAMP) is an isothermal PCR amplification technique that can be performed in peripheral health care settings. The LAMP-based TB-LAMP assay (Eiken Chemical Company, Tokyo, Japan) has been recommend by WHO as a potential replacement for smear microscopy since 2016, owing to its superior diagnostic performance. It also does not require much sophisticated laboratory equipment (16) (Table 1). Despite this, TB-LAMP is underutilized (17), but some countries are creating their own LAMP assays for in-country use. Hopefully country-specific versions of LAMP will increase uptake.

### Next-generation Xpert testing.

In 2010, WHO endorsed Xpert MTB/RIF use with the GeneXpert platform (Cepheid, Sunnyvale, USA (18), and an updated policy was released in 2013 (19). In 2017, WHO recommended Xpert Ultra (Cepheid) (Ultra), the next generation of Xpert MTB/RIF, as the initial TB diagnostic test for adults and children, regardless of HIV status, over smear microscopy and culture (20). As in previous generations, Ultra detects RIF resistance by employing four probes with targets in the *rpoB* gene and melting temperature analysis (Table 1). Compared with previous generations, Ultra test cartridges have a larger chamber for DNA amplification than Xpert MTB/RIF and two multicopy amplification targets for TB, namely, *IS6110* and *IS1081*, for a lower limit of detection of 16 CFU/ml. These modifications have increased Ultra’s overall sensitivity from 85% (95% confidence interval [CI], 82% to 88%) to 88% (95% CI, 85% to 91%); however, compared with the previous generation, Ultra’s specificity is lower at 96% (95% CI, 90% to 98%) versus 98% (95% CI, 97% to 98%), seemingly because it detects nonviable bacteria, particularly in people with recent TB (21, 22). This lower specificity is proving to be an important issue in certain settings, such as areas with high numbers of HIV-TB coinfections or recurrent TB cases, like South Africa. In a recent study by Mishra and colleagues, it was shown that the Xpert Ultra assay had significantly lower specificity and positive predictive value than the Xpert MTB/RIF assay and high numbers of Ultra positive/culture negative people with previous treatment (23). The clinical consequences of treating such patients are unclear, and ongoing studies are attempting to shed light on this information.

The Xpert Ultra test also has a semiquantitative “trace” category, indicating bacilli at the lowest limits of detection. In instances of trace positives (termed “trace calls”), one of the two multicopy amplification targets, but not the *rpoB* sequences, are detected. In instances of suspected extrapulmonary TB, children, and people living with HIV (PLHIV), trace positives should be treated as positives, as these cases tend to be paucibacillary. For other cases, a fresh specimen should be retested to rule out false positives (20). Trace calls may be difficult to interpret, as in the aforementioned study by Mishra et al., where it was observed that among people who were previously treated for TB, trace positives were a substantial portion of all positives, and these individuals by definition had indeterminate results for RIF resistance and were culture negative, precluding further DST (23). Trace calls may be improving Ultra’s sensitivity for extrapulmonary TB, particularly in the context of definite or probable TB meningitis, where a sensitivity of 70% (95% CI, 47% to 87%) in cerebrospinal fluid was observed (24); however, this finding is not consistent across studies, as another group observed a sensitivity of definite or probable TB of only 49% (95% CI, 35% to 63%) (25). Notably, even with sensitivity of 77%, as observed in another study of TB meningitis (26), the Ultra test’s negative predictive value is still too low for use as a rule-out test. Research on Ultra for TB lymphadenitis (27) has shown sensitivities of 70% using fine-needle aspiration and 67% using tissue biopsy in a study of 99 people with suspected TB lymphadenitis (27). In a multisite study using 317 frozen pleural fluid samples, Ultra sensitivity was 44%, compared with that of the Xpert test at 19% (28). More research will be necessary to determine if Ultra’s performance for other forms of extrapulmonary TB has improved over the Xpert MTB/RIF assay (29).

As an automated PCR-based test, Ultra can be used by minimally trained technicians, but as it runs on the GeneXpert platform, it requires a continuous power supply and computer which limits its use as a true point-of-care (POC) test. Alternatively, the recently launched GeneXpert Edge system is battery powered and utilizes a tablet, making it more portable.

### Made in India: Truelab by Molbio.

Truenat MTB, Truenat MTB Plus, and Truenat MTB-Rif Dx (Molbio Diagnostics, Goa, India) are chip-based, micro real-time PCR-based assays for TB detection that produce results in 1 hour on the portable Truelab platform (Molbio Diagnostics). Already being rolled out in India, Truenat is characterized as a more affordable alternative to Xpert that is made in India. Products that are developed and manufactured in a country with a high TB burden might be quicker and more straightforward to scale up in that country than products developed in another country, as governments often already have a degree of buy-in, data from locally run studies will have accumulated, and supply chain and regulatory issues are simpler to solve (30, 31).

Truenat MTB and Truenat MTB Plus assays detect M. tuberculosis bacilli in sputum after extraction using the separate TruePrep instrument and kits, with Truenat MTB-Rif Dx available as an optional add-on chip for sequential RIF resistance detection (32). Truelab, which comes in Uno-, Duo-, and Quattro-throughput formats, was designed to be “rugged” and POC friendly, as it has a dust filter and runs in temperatures up to 30°C, but multiple micropipetting steps necessitate a trained technician for its operation.

In December 2019, WHO convened a guideline development group meeting to determine recommended use cases for Truenat assays and other rapid molecular tests. The subsequent rapid communication reported that Truenat MTB, MTB Plus, and MTB-Rif Dx assays displayed comparable sensitivities and specificities to Xpert MTB/RIF and Ultra for the detection of TB and RIF resistance, although this report was based on an interim analysis of a multicenter study that is still ongoing. The 2020 WHO Consolidated Guidelines on Molecular Diagnostics recommend using Truenat MTB or MTB Plus rather than smear microscopy as an initial diagnostic test for TB in adults and children with signs and symptoms of pulmonary TB. This is a conditional recommendation, as test accuracy certainty is moderate. Regarding DST, with a Truenat MTB- or MTB Plus-positive result, Truenat MTB-RIF Dx may be used as an initial test for rifampicin resistance rather than phenotypic DST. This is also a conditional recommendation, as there is very low certainty of evidence for test accuracy (21).

## EMERGING TECHNOLOGIES

### Xpert XDR.

Another PCR-based cartridge has been designed to run on the GeneXpert and Omni platforms for the simultaneous detection of mutations associated with resistance to multiple first- and second-line TB drugs or extensively drug-resistant TB (XDR-TB). Against phenotypic drug-susceptibility testing, a prototype version of the Xpert XDR cartridge displayed sensitivities (95% CI) of 83.3% (77.1% to 88.5%) for isoniazid, 88.4% (80.2% to 94.1%) for ofloxacin, 96.2% (87.0% to 99.5%) for moxifloxacin at a critical concentration of 2.0 μg per milliliter, 71.4% (56.7% to 83.4%) for kanamycin, and 70.7% (54.5% to 83.9%) for amikacin (33). In July 2020, the Xpert MTB XDR-TB cartridge was launched, but further validation and WHO review are pending (85). As WHO updates treatment guidelines for MDR-TB and XDR-TB, it will be critical that molecular tools for DST can be updated to quickly reflect new recommendations. Already, this iteration of Xpert XDR may have less impact than it otherwise would have, as WHO has de-emphasized second-line injectable agents for treating drug resistant forms of TB (34). Future developments will need to focus on drugs that are now critical for MDR and XDR-TB management, including bedaquiline, pretomanid, and linezolid (35), but developing highly accurate molecular diagnostics to detect resistance to these drugs is currently impossible due to the lack of knowledge on resistance mechanisms.

### GeneXpert Omni and other point-of-care devices.

The GeneXpert platform was originally designed for use at the district or subdistrict level. Although efforts were made to use the technology at lower tiers of the health system, it soon became evident that microscopy centers in countries with a high TB burden often lacked the infrastructure necessary for this technology, including continuous power and temperature controls (10). As such, the POC GeneXpert Omni platform is a long-awaited development, as it will permit the use of Xpert MTB/RIF and Ultra assays in decentralized locations (e.g., primary care centers). Although delays have pushed back its launch repeatedly, Omni promises to be a real POC platform with a 2-day battery life and no tablet or computer requirement (36). The first instruments will be available in 2021, and Omni will eventually be able to run Ultra and any other Xpert cartridges that become available.

Other such POC NAATs are also under development. For example, Q-POC from QuantuMDx (Newcastle-upon-Tyne, United Kingdom) is a POC battery-operated PCR system that promises to deliver TB testing results in less than 30 min. It has been evaluated in combination with oral swabs as a sample, where its sensitivity and specificity, in preliminary studies, were similar to that of Xpert (37).

### Indigenous Chinese diagnostics.

Similar to Molbio in India, Chinese biotechnology firms have used their own expertise to develop TB NAATs for in-country use. These companies have undergone the China Food and Drug Administration (CFDA) regulatory processes, received approval, and rolled out the tests nationally. However, none of these technologies have been reviewed by WHO, and therefore, uptake by other countries is limited. Table 2 summarizes the performance of some of these assays from systematic reviews (38, 39).

**TABLE 2:** CFDA-endorsed molecular test for TB diagnosis and drug susceptibility testing^*a*^

Technology	Method principle	Intended use	Sensitivity (%)	Specificity (%)	Target setting of use	Reference
EasyNAT	Cross priming amplification	M. tuberculosis diagnosis	87 (pooled)	97 (pooled)	District or subdistrict laboratory	38
SAT-TB	Isothermal amplification of M. tuberculosis 16S RNA	M. tuberculosis diagnosis	71–94 (range)	54–83 (range)	District or reference laboratory	38
MeltPro TB	PCR, melt curve analysis	DST	98 (RIF resistance), 85 (INH resistance), 64 (FLQ resistance), 83 (SLID resistance)	97 (RIF resistance), 98 (INH resistance), 98 (FLQ resistance), 99 (SLID resistance)	Reference laboratory	39
GeneChip MDR	PCR, hybridization	MDR-TB diagnosis; INH and RIF resistance	79 (MDR-TB), 89 (RIF resistance), 79 (INH resistance)	98 (MDR-TB), 97 (RIF resistance), 97 (INH resistance)	Reference laboratory	39

a CFDA, China Food and Drug Administration; DST, drug susceptibility testing; INH, isoniazid; RIF, rifampin; SLID, second-line infectible drugs.

CFDA-approved since 2014, EasyNAT (Ustar Biotechnologies, Hangzhou, China) replicates and detects mycobacterial DNA from sputum via cross-priming amplification (CPA). As CPA is an isothermal technique, EasyNAT may be placed at low levels of health care systems, as a thermal cycler is not required (38). A fully integrated and automated next-generation version is in development (40).

Simultaneous amplification and testing TB (SAT-TB) (Rendu Biotechnology, Shanghai, China) detects mycobacterial 16S rRNA from sputum, which is isothermally amplified before the resultant cDNA is detected by fluorescent probes, requiring laboratory infrastructure, such as adequate biosafety facilities for specimen manipulation and trained personnel (41).

For drug resistance testing, MeltPro TB (Zeesan Biotech, Xiamen, China) assays for RIF, INH, second-line injectables, and fluoroquinolones are available, allowing them to detect MDR-TB and XDR-TB. After manual DNA extraction, MeltPro TB detects drug resistance via melt curve analysis using a PCR machine; the shift in melting temperature from wild type to mutation in sequences covered by multiple probes can be qualitatively detected (42).

GeneChip MDR (CapitalBio Corporation, Beijing, China) is a microarray assay that requires hands-on sample preparation before reverse hybridization and analysis on a fully automated system. As such, it requires sophisticated laboratory equipment. GeneChip MDR utilizes multiplexed asymmetric PCR to detect resistance to RIF and INH in one assay and thus can detect MDR-TB (43).

### High-throughput solutions: centralized diagnostic tests.

Recently, centralized, high-throughput NAATs for TB diagnosis and drug resistance detection have been developed and are currently undergoing WHO evidence evaluation. RealTime MTB (Abbott Molecular, Abbott Park, USA), RealTime RIF/INH (Abbott Molecular), FluoroType MTB (Hain Lifescience, Nehren, Germany), FluoroType MTDBR (Hain Lifescience), Cobas MTB (Roche, Rotkreuz, Switzerland), and Max MDR-TB (BD, Franklin Lakes, USA) assays run on established multidisease platforms that are already employed for such diseases as human immunodeficiency virus (HIV), human papillomavirus, and hepatitis C virus (44). These almost entirely automated tests are all intended for tertiary laboratory use. In 2019, a WHO technical expert group meeting reported that the centralized assays’ performance for detecting resistance to INH and RIF was similar to LPA and that RealTime MTB, Cobas MTB, and Max MDR-TB performed similarly to Xpert MTB/RIF for TB detection (45). For now, these assays are recommended for operational research use only, with a WHO review of broader use expected in late 2020.

The RealTime MTB is a multiplex NAAT that targets the MTB *IS6100* and *PAB* genes with a limit of detection (LOD) of 17 CFU/ml. Up to 96 respiratory specimens can be inactivated and processed by the Abbott m2000 platform per run (46). A systematic review and meta-analysis of 10 studies incorporating 4,858 specimens found that RealTime MTB had a sensitivity of 96% (95% CI, 90% to 99%) and specificity of 97% (95% CI, 94% to 99%) for TB detection; regarding RIF resistance detection, it had a pooled sensitivity of 94% (95% CI, 89% to 99%) and specificity of 100% (95% CI, 99% to 100%); and for INH resistance detection, its pooled sensitivity was 89% (95% CI, 86% to 92%) and specificity was 99% (95% CI, 98% to 100%) (44).

Another centralized test is the semiautomated FluoroType MTB, a beacon-based PCR assay performed on the Hain Fluorocycler platform. Specimen decontamination, sample preparation, and DNA isolation must be performed manually, which requires 30 min of hands-on time, with the entire process taking 4 h to final results (47). In a systematic review and meta-analysis of five studies incorporating 2,660 specimens, FluoroType MTB displayed a sensitivity of 92% (95% CI, 88% to 93%) and specificity of 99% (95% CI, 64% to 100%) (44).

The Cobas 6800/8800 MTB assay runs on the high-throughput Cobas 8800 platform that can run 960 samples in 8 h. One internal manufacturer study of 744 samples reported a sensitivity and specificity of 95% (95% CI, 92% to 97%) and 98% (95% CI, 96% to 99%), respectively (48).

Finally, the Max MDR-TB test runs on the BD Max platform and targets the MTB 16S rRNA gene. Up to 24 specimens are manually decontaminated and prepared before extraction and amplification by the Max MDR-TB assay. Time to final results is 4 hours (49). A manufacturer-sponsored validation study of 892 samples reported TB detection sensitivity of 93% (95% CI, 89% to 96%) and specificity of 97% (95% CI, 96% to 98%). Sensitivity for RIF resistance and INH resistance was 90% (95% CI, 55% to 100%) and 82% (95% CI, 63% to 92%), respectively, with 100% specificity in both cases (50).

Centralized TB assays are promising due to their high diagnostic accuracy and ability to run large numbers of samples simultaneously, and their automated nature reduces the hazard of contacting infectious respiratory specimens for health care workers and laboratory technicians. The developmental pipeline for centralized assays is quite robust, with platforms, such as MeltPro (Zeesan Biotech, Xiamen, China), Seegene (Seoul, South Korea), and MolecuTech (YD Diagnostics, Seoul, South Korea), currently under regulatory assessment (51). All platforms are offering tests for MDR-TB and XDR-TB, which will provide more options in the future.

However, carry-over contamination is still possible with these assays, and quality assurance is critical. Additionally, the costs for each of these tests have not been made public, and no subsidized or concessional pricing schemes are yet in place. These tests do run on multidisease platforms, which adds value, but it is unclear exactly who will be willing to pay to implement these tests if they can only perform DST for INH and RIF resistance, particularly when there are simpler NAATs available (Table 1). Furthermore, their centralized placement means they are unavailable where patients first present to care, and therefore, sample transportation is essential for success. Reliable systems for delivering test results to patients and health care providers must also be in place for these tests to have impact.

### Next-generation sequencing.

Next-generation sequencing (NGS) is increasingly considered a promising option for comprehensive DST for TB and produces results much faster than traditional phenotypic culture or culture-based testing (52, 53). Unlike probe-based assays where detection is limited to probe-specific targets, NGS-based assays can provide detailed and accurate sequence information for whole genomes, as with whole-genome sequencing (WGS), or multiple gene regions of interest, as with targeted NGS (54). (Table 3).

**TABLE 3:** Strengths and limitations of WGS versus targeted sequencing via next-generation sequencing

Whole-genome sequencing	Targeted sequencing
Strengths	Strengths
Full genome sequenced	Sequence directly from sample
No prespecified targets needed	Large number of gene targets
Comprehensive solution	Less expensive than WGS
Detect rare mutations and heteroresistance	Simpler bioinformatics and storage
	Detect rare mutations and heteroresistance
Weaknesses	Weaknesses
Requires culture isolates	Knowledge of targets required
Slower than targeted NGS	Less information than WGS
Complicated bioinformatics	Expensive
Expensive	

Acknowledging the value of NGS, WHO has published guidance on the role of sequencing for detecting mutations associated with drug resistance in TB (54), along with a consensus-based TPP for sequencing. In 2019, a TB sequencing database called ReSeqTB was established at WHO to curate, standardize, and unify genotypic and phenotypic DST data, along with metadata on drug-resistant TB (DR-TB) (55).

**FIG 2:**
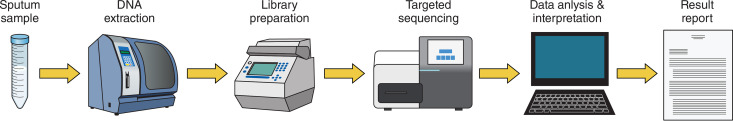
Targeted sequencing workflow schematic.

There are ongoing efforts by multiple stakeholders to validate targeted sequencing as a complete end-to-end solution for DR-TB detection, from DNA extraction direct from respiratory samples (i.e., without the need for first culturing and then isolating a specimen), targeted library preparation and sequencing, to result reporting (Fig. 2). One such targeted assay that is currently available in the market is Deeplex Myc-TB (Genoscreen, Lille, France). Deeplex Myc-TB uses ultradeep sequencing of 24-plex amplicon mixes for mycobacterial species identification, genotyping, and DST. In addition, the manufacturer indicates that it can detect heteroresistance, i.e., the phenomenon of subpopulations within a seemingly uniform microbial population displaying both resistance and susceptibility to a particular drug (56), down to 3% of minority strains in cases of multiple infections or emergent mutations (57). Another newly developed targeted sequencing assay for DR-TB is DeepChek-TB (Translational Genomics Research Institute, Flagstaff, USA), which has recently been licensed by ABL (Luxembourg) (58). Both tests are currently for research use only.

Sequencing is currently being successfully implemented for DR-TB surveillance purposes in at least seven countries—Azerbaijan, Bangladesh, Belarus, Pakistan, Philippines, South Africa, and Ukraine (59). Select health systems in setting with low TB burden, including the United Kingdom (Public Health England), the Netherlands, and New York state, have already transitioned from phenotypic culture to WGS for DST for first-line drugs (60, 61). The US Centers for Disease Control and Prevention sequence isolates from all culture-confirmed TB cases nationwide (62).

More countries are considering switching to a sequencing-based approach for the surveillance of drug susceptibility. For example, India has recently expressed interest in utilizing sequencing for surveillance and clinical care. In 2018, infrastructure and technical support for sequencing were introduced at five national TB program laboratories across India with Global Fund funding. It is hoped that this will be the beginning of the foundations of a clinical diagnostic network in the future (63).

South Africa has implemented and integrated sequencing into their national drug resistance surveillance program as an alternative to phenotypic DST and are considering its future potential for laboratory-based TB management and TB transmission investigations (64).

In Brazil, the interdisciplinary group Rede Brasileira de Pesquisas em Tuberculose (REDE-TB, Brazilian TB Research Network) identified NGS as a key technology for implementation. Through the Oswaldo Cruz Foundation (Fiocruz), Brazil has also signed memoranda of understanding with the Beijing Genomic Institute and the Chinese Centre for Disease Control and Prevention. One of the planned activities under this agreement is the establishment of a sequencing service at Fiocruz with applications in infectious disease, including TB (65).

Regarding sequencing for DST, centralized sequencing platforms have been the norm, but there is increasing interest in smaller and more portable sequencing devices, such as MinION (Oxford Nanopore, Oxford, UK) (66) and iSeq from Illumina (San Diego, USA) (67), and validation for both is on-going.

### Potential for integrating NAAT testing for TB and COVID-19.

Across the world, health care systems are being upended by the COVID-19 pandemic, but it is critical we do not neglect other diseases like TB that persist outside the spotlight (68, 69). A modeling study suggests that one unintended result of the pandemic-related lockdowns is a projected 1.5 million excess TB-related deaths from 2020 to 2025 (3). Countries must act now to ensure that routine care for patients experiencing other disease continues and to ensure these projections do not become reality.

One clear area for intervention is the integration of TB and COVID-19 testing. As patients with either disease may present with cough, fever, or difficulty breathing, this represents an opportunity to test presumptive patients for TB and COVID-19 in one clinical encounter. This dual testing would be more convenient for patients and health care workers, as it could reduce the number of necessary follow-up visits.

The recently launched Xpert Xpress SARS-CoV-2 (Cepheid, Sunnyvale, USA) cartridge might allow low- and middle-income countries (LMICs) to increase their capacity to test for COVID-19, as many countries already have existing GeneXpert networks (70). However, concern has been expressed that a ramp-up of COVID-19 testing on the GeneXpert system may come at the expense of TB testing in LMICs that rely on Xpert MTB/RIF (3, 68). Abbott and Roche also have released COVID-19 assays to run on their centralized testing platforms, namely, RealTime (71) and Cobas 6800 (72), respectively. Both systems are used in some reference laboratories of countries with high TB burden for multidisease testing. In India, Molbio has released a COVID-19 test for the Truelab platform (73) that is now in use.

Leveraging existing multidisease NAAT platforms for both TB and COVID-19 testing could be an effective strategy. Research into using a common respiratory sample (e.g., sputum) will be necessary to understand the feasibility of this strategy and to address biosafety concerns.

### Conclusion: optimizing the impact of NAATS.

Advances in molecular TB diagnostics in the last decade have resulted in TB tests that are highly accurate and faster than conventional microbiological tests, and emerging technologies promise to continue this trend. In some respects, NAATs are having a positive clinical impact. For example, it has been shown that routine use of the Xpert test leads to reductions in time to TB diagnosis and time to treatment initiation, from several days to same-day (4, 5, 74–76), and its use also facilitated increased numbers of patients to commence anti-TB treatment (5, 76). However, for long-term outcomes like mortality, NAAT impact is more ambiguous (77, 78), albeit inherently difficult to measure appropriately (79).

As long as cascades of care in high TB settings remain weak or fragmented, diagnostic testing alone will be unable to decrease mortality or disease recurrence, and evaluations of NAAT clinical significance will continue to produce null results (80). Issues such as underutilization of existing NAATs, empirical treatment of people with suspected TB, and patient loss to follow-up all reduce the potential beneficial effect of diagnostic testing (81). Thus, optimizing the clinical impact of molecular tests for TB will require their introduction into functioning, strengthened health care systems, which can also respond to outbreaks that require multidisease testing capacity (82). Centering patients within high-quality health systems will allow NAATs to reach their full potential and become an integral part of a digitally connected, patient-centered, reimagined TB care system (83).
